# Microscopy of the Teeth

**Published:** 1868-05

**Authors:** S. P. Cutler

**Affiliations:** Holly Springs, Miss.


MICROSCOPY OF THE TEETH.—BY S. P. CUTLER, M. D., D. D. S., HOLLY SPRINGS, MISS.—I do not propose giving anything particularly new in the anatomical structure or organism of the Dental structures of the human teeth. The department of microscopic histology of the teeth has been extensively investigated by Retzius, Nasmyth, Owen, Tomes, Kölliker, Prof. Leidy, and many other able microscopists, with superior instruments, which leaves but little if anything to be added that is not already known, though I do not regard the subject as exhausted. It is like the golden sands that have been carefully washed over and over again; still some shining particles may be discovered which had hitherto escaped observation. I propose giving the results of my own observations and my own views in relation to the anatomy, physiology, and pathology of the human teeth after their development.

My observations have been made upon sections from every portion of the teeth of different ages.

The teeth, as is well known, are entirely different in their anatomical structure, as well as their functions and uses, from any other bony structure of the animal system.

The bones constituting the frame-work of the system, giving form and action in motion, furnishing cavities for the vital organs, are all of them covered, and, where hollow lined with a membrane which furnishes nutrition to them, and, in case of disease or injury, aids in their restoration.

On the contrary, the teeth are not susceptible of any vital restoration after injury or disease, with only a single exception — that is, of exostosis of nerve cavity resulting generally from wearing away crown by natural use or friction, which will be noticed under the head of physiology and pathology, where it properly belongs.

When these organs have been once fully developed, they come to a stand-still state, or in other words, a state of inertia.

The crowns of teeth have no animal covering, but, on the contrary, have a hard, adamantine covering, almost entirely composed of rock-like substance, chiefly phosphate of lime.

A tooth consists of a crown, neck, and fang, or root, occupied in the interior by a nerve pulp, membranes, and a system of blood-vessels. The membrane passing out at the apex of the fang furnishes covering to the fang, and attachment to the alveolus or socket. The surface of the fang under this membrane is covered by a substance somewhat different from dentine, which constitutes the body of the tooth, called crustæ petrosæ, and terminates after covering every portion of the fang at the neck, where the enamel meets it under the margin of the gum, which is adherent to the neck, except the loose margin which covers the beveled edge of the enamel.

It would be out of place here to speak of articulation of the teeth, or of their uses, as that subject is thoroughly understood by all intelligent members of the profession.

The ivory or dentine which forms the body of the tooth is white, dense bone, harder and more compact than any other bony structure, over two-thirds of which is composed of salts of lime, the balance of fibrous or animal substance.

This structure is made up of a system of capillary tubes, called tubuli, exceedingly small, ten thousand of which, placed side by side, in contact, would measure only one inch ; so estimated by Carpenter, Quickett, and other microscopists.

The tubes commence at the nerve cavity, with open mouths, and radiate outwardly, never losing themselves or ending until reaching the enamel in the crown and crustiœ petrosæ, and membrane in the neck and fang, with exceptions in the latter.

Specimens properly prepared present under high powers of the microscope one of the finest views imaginable; spread out on a fifteen-inch field, presents at one view many hundreds of tubuli.

The tubuli leave the pulp cavity nearly at right angles from all parts — portions of which they leave at perfect right angles, other portions slightly oblique, in the direction of cutting or grinding surface. All those that leave the nerve cavity at right angles generally pass in a direct line without any curve, and meet the surface at right angles. In molar teeth, in the crowns alone there are four or five points where the tubuli leave and terminate at right angles — that is, directly over the point of each corner or horn of the pulp, and under the centre of the grinding surface or centre of the depression or fossa.

From the sides of the pulp cavity the tubuli leave nearly at right angles, but soon after leaving the cavity they begin to curve upward for a certain distance, then mounting upward and outward for a given distance, they continue in a more or less direct line or straight course; then commence and curve outward just before reaching the enamel, or just before the tubuli branch or bifurcate this second curve, nearly corresponding to the primary curve, the two curves forming a perfect O. G. figure. The inner curve is generally more sudden than the outer curve. The outer curve is generally longer, sometimes the reverse. These curves are generally very regular. Where the tubes are longest, the largest O. G., and where shortest, the shortest figure.

In some instances the secondary curve commences where the first one leaves off — that is, where the distance is least from point to point, and where longest there is considerable space where the tubes are straight between the primary and secondary curves.

At some points there is minor curve upward just at or before entering the enamel; this is oftener seen in the molars just below the cusps than anywhere else.

All around the cornu or horn of the pulp, whether it be in teeth that have four or only one cusp, the tubuli, after leaving the cavity, suddenly curve upward, and continue upward in a parallel course or nearly so with those that leave the cavity directly over the tip, and of course, as before stated, forming in case of side tubes the O. G. These are generally the longest of any tubuli. The tubuli in the grinding surface of molars are already described in the above explanation, which will readily be understood by studying the form of a molar.

The above equally applies to incisors, cuspidati, and bicuspids,— one explanation applies to all.

The tubuli from the sides of the nerve cavity in the neck mount upward, and most of them reach the enamel.

The object of this seems to be to bring all that can possibly be brought in contact with the enamel and to fill up and occupy the crown of the tooth.

This explanation accounts for the curvatures of the tubuli. Should these tubuli all pass directly out at right angles in a direct course to the neck of the tooth, a more extensive branching of tubuli would be requisite to fill up completely all portions of the crown substance which would at the same time give an undue amount of sensibility to the neck of the tooth which is not protected equally by enamel, and would be more likely to give trouble. The above reasons and explanations may not be beyond criticism, but will probably be received and adopted by the profession until better ones are brought forward to take their place.

The tubuli are hollow tubes open at one end and closed at the other, without any valvular or any other apparent obstruction during their whole course. These tubuli continue the same size from nerve cavity to the point where the first branch leads off, and from that point to the next branch both branches are of equal size, and these branches just half the size or capacity of the tubuli up to that point, and so from nerve cavity to enamel, wherever branching takes place, and where there is no branching there is no diminution in tubuli.

Where the tubuli are strictly parallel in adult teeth, no primary branching or bifurcations are to be found. Neither is there any gradual taper discoverable like a tree, as I see represented in the *Dental Cosmos* from a drawing by Prof. Leidy.* The branchings in the primary tubuli are generally

*The illustration objected to, as a *diagram*, is in general accordance with *nature*, and the illustrations presented in the works of Retzius, Nasmyth, Tomes, etc.—J. H. M'Q.

simply bifurcations; in some instances, however, three forks are discoverable at precisely the same point, though not frequent only at their terminal branching. These three branches, where they exist, have the capacity of the trunk that formed them as before stated.

The system of tubuli cannot be regarded as exclusively dichotomous, as stated by microscopists, but a mixed one of both di- and trichotomous both in their general and terminal branchings.

The tubuli are apparently in specimens where two or more thicknesses are left, regular even lines, both on thinning the specimens to a solitary thickness they are found to be full of short, irregular crooks, during their whole course. These short crooks are more apparent under high powers. Notwithstanding the tubuli are full of short irregular crooks, they never at any point in their course touch each other or come in contact.

The object of branching of the tubules seems to be to fill up space where there is a divergence.

The surface of the nerve cavity being smaller than the surface of the tooth, there must necessarily be more or less branching in order to make the intertubular spaces about equal throughout, otherwise there would be a greater predominance in the intertubular spaces toward the circumference.

Just before the tubules enter the membrane that exists between the enamel and dentine they, every solitary one of them, branch or bifurcate, and in many instances trifurcate; the sum of the capacity of these branches, as already stated, being just equal to the trunk that formed them. These branches diverge generally at an angle between 50° and 75°, and cross each other in different directions as they enter the membrane which might be denominated coronal or inter-enamel membrane. The terminal branchings of tubuli form a universal delta just before entering the enamel and when taken collectively form a beautiful coronal arch.

The intertubular spaces, or the spaces between the tubes, are greater than the diameter of the tubes,—in other words, double the number of tubes could occupy the same spaces without actually coming in contact. There seems to be no regularity in the tubular passage through the intertubular spaces.—*Dental Cosmos.*

